# PRACT-India: Practical Recommendations on Acne Care and Medical Treatment in India—A Modified Delphi Consensus

**DOI:** 10.3390/antibiotics14080844

**Published:** 2025-08-20

**Authors:** Nina Madnani, Abir Saraswat, Anand Nott, Deepak Jakhar, Lalit Kumar Gupta, Malavika Kohli, Manas Ranjan Puhan, Prabhakar Sangolli, Rahul Nagar, Sanjay Kumar Rathi, Sanjeev Aurangabadkar, Satish DA, Seetharam KA, Sunil Dogra, Dhiraj Dhoot, Ashwin Balasubramanian, Saiprasad Patil, Hanmant Barkate

**Affiliations:** 1 Department of Dermatology, Sir PD Hinduja Hospital and Medical Research Centre, Mumbai 400016, India; 2 Indushree Skin Clinic, Lucknow 226016, India; 3 Skin Research Centre, Chennai 600090, India; 4 Dermosphere Clinic, Delhi 110075, India; 5 Department of Dermatology, RNT Medical College, Udaipur 313004, India; lalitanj@yahoo.com; 6 Skin Secrets Clinic, Mumbai 400093, India; 7 Niki Skin Care, Bhubaneshwar 751013, India; 8 Department of Dermatology, Sri Siddhartha Institute of Medical Sciences, Benagluru 562123, India; 9 Department of Dermatology & Venereology and Leprosy, Mahatma Gandhi Memorial Medical College, Indore 452001, India; 10 Dr. Rathi’s Skin Clinic, Siliguri 734001, India; 11 Aurangabadkar’s Skin & Laser Clinics, Hyderabad 500095, India; 12 Department of Dermatology, Sagar Hospitals, Bengaluru 560041, India; 13 Department of Dermatology, Venereology & Leprosy, GSL Medical College, Rajahmundry 533296, India; 14 Department of Dermatology, Venereology & Leprosy, Postgraduate Institute of Medical Education & Research (PGIMER), Chandigarh 160012, India; 15 Department of Global Medical Affairs, Glenmark Pharmaceuticals, Mumbai 400099, India

**Keywords:** acne, consensus, antibiotics, cosmeceuticals, dermatitis, lactation, pregnancy, retinoids

## Abstract

**Background/Objectives:** Acne vulgaris is a prevalent dermatological condition, yet clear, region-specific management guidelines, particularly for India’s diverse population, remain limited. Effective acne management extends beyond pharmacologic therapy, emphasizing proper skincare, patient education, and adherence strategies. This consensus aims to provide tailored, evidence-based recommendations for optimizing acne treatment in the Indian context. **Methods:** A panel of 14 dermatology experts with ≥15 years of experience reviewed literature, real-world clinical practices, and patient-centric factors relevant to acne management in India. Using a modified Delphi process with two virtual voting rounds, 61 statements across seven clinical domains were evaluated. Consensus was defined as ≥75% agreement. **Results:** Topical retinoids remain the first-line therapy, with combination regimens (benzoyl peroxide or topical antibiotics) preferred to enhance efficacy and minimize antibiotic resistance. Hormonal therapies, including combined oral contraceptives and spironolactone, are recommended for females with resistant acne. Guidance includes individualized treatment plans, baseline investigations, and selection of appropriate topical and systemic agents. Special considerations for pregnancy and lactation prioritize maternal and fetal safety. **Conclusions:** This expert consensus provides practical, evidence-based recommendations for acne management in India, integrating pharmacological and non-pharmacological approaches. The tailored guidance supports individualized care, antibiotic stewardship, and improved treatment adherence, aiming to enhance patient outcomes nationwide.

## 1. Introduction

Acne vulgaris is a chronic condition of the pilosebaceous unit [1]. It is multifactorial in origin, most commonly presenting during adolescence but often persisting into adulthood [2]. The global prevalence is estimated at 20%, while in Asia, it is slightly lower at 19.4% [3]. In India, acne predominantly affects individuals aged 18–25 years, with a significantly higher prevalence in females (81.7%) [2,4].

Diagnosis is based on identifying characteristic lesions, which include noninflammatory comedones (open or closed) and inflammatory lesions such as papules, pustules, and nodules [1]. Adolescent acne is more straightforward to diagnose, whereas adult-onset acne may require endocrine evaluation [5]. Common sequelae include facial scarring, postinflammatory erythema (PIE), and postinflammatory hyperpigmentation (PIH), which can often be more distressing than the acne itself [2,4,6]. Severe acne and scarring are frequently associated with a positive family history [7]. An observational study conducted in South India reported scarring in 34% of cases and pigmentation in 40%, with a family history of acne present in 33% of the individuals [8]. The risk of scarring is further increased in patients with skin-picking behaviors or excoriation disorder [9]. Delayed treatment initiation is another key contributor to scarring [10]. As a result, patients with active or unresolved acne frequently experience psychological distress, including depression and negative body image [11].

Standardized acne severity assessment is critical for guiding treatment and evaluating outcomes. Common tools include the Global Acne Grading System (GAGS), the Physician Global Assessment (PGA) score, the Leeds Revised Acne Grading, and the Truncal Acne Severity Scale (TRASS) [12]. Effective acne management requires a multidisciplinary, patient-centered approach that addresses pathogenesis, clinical presentation, and individual preferences [4]. Topical retinoids remain the mainstay of therapy but may cause retinoid-induced dermatitis, necessitating careful use [13]. Dermocosmetics, or cosmeceuticals, are skincare formulations with active ingredients that combine cosmetic and therapeutic benefits [14]. These agents can serve as monotherapy in mild cases or can be used adjunctively in moderate-to-severe cases, enhancing both tolerability and adherence [15,16]. A structured skincare routine, particularly with effective cleansing agents, may augment therapeutic outcomes [17]. Patient education is essential for treatment success. Clinicians should discuss the chronic nature of acne, expected timelines, appropriate medication use, and the important of maintenance therapy. Understanding patient perspectives and tailoring treatments suited to their lifestyle can significantly improve adherence [18].

Evidence on acne management specific to the Indian subcontinent remains limited. This expert consensus seeks to address key gaps:

1. Limited Indian data, with most existing research based on Western populations and possibly not reflecting Indian clinical and epidemiological nuances.
2. A lack of comprehensive national guidelines that incorporate newer therapies and recommendations for special populations.
3. Insufficient practical guidance to support real-world clinical decision-making.
4. Unique clinical challenges in managing acne during pregnancy and lactation.

This consensus provides practical, evidence-based recommendations on the management of acne vulgaris to dermatologists and general physicians in India, including diagnosis, therapeutics, and special considerations such as care during pregnancy and lactation.

## 2. Results

Of the 61 clinical statements proposed, eight did not reach the predefined threshold of 75% agreement in the first round and were subsequently revised and discussed further. By the end of round two, 36 statements had been finalized. This iterative modified Delphi process ensured that the final recommendations reflected a balanced and evidence-informed expert consensus. The panel achieved agreement on several key aspects of acne vulgaris management in the Indian context, with an emphasis on individualized, patient-centric care. The finalized recommendations are presented in Table 1.

## 3. Discussion

The expert discussion explored key findings on acne management, highlighting the efficacy and safety of various treatment options while addressing research gaps. The discussion below highlights key aspects of acne management, including treatment efficacy, challenges, and special considerations.

### 3.1. Baseline Investigations and Screening in Acne Vulgaris

Recommendations 1–5 addresses essential baseline investigations for individuals with acne vulgaris. Testing for serum triglycerides and alanine transaminase (ALT) is advised prior to initiating isotretinoin, given its known effects on lipid metabolism and liver function [13]. However, for patients with normal baseline values, routine repeat testing may not be necessary [43].

Culture and sensitivity testing in acne is typically reserved for severe, atypical, or treatment refractory cases or where secondary infection is suspected [44]. In female patients, particularly those with late-onset or treatment-resistant acne, hormonal evaluation is strongly recommended. A study reported that 45% of female acne patients who underwent pelvic ultrasound and blood testing were diagnosed with polycystic ovary syndrome (PCOS), highlighting the clinical importance of endocrinological evaluation in this population [45,46]. Insulin resistance, another metabolic factor, is also associated with acne pathogenesis and may warrant assessment [47]. For females of reproductive age, a pregnancy test is imperative prior to initiating isotretinoin due to its well-established teratogenic risks [20]. Additionally, while isotretinoin has been associated with rare psychiatric side effects, such as depression, psychological screening may be considered on a case-by-case basis [21].
▪***Key expert recommendations***▪ Psychological screening may be selectively performed in patients initiating isotreitnoin.▪ Hormonal work-up, including serum androgens, fasting insulin, LH/FSH, prolactin, and pelvic ultrasound, is advised in patients with persistent or treatment-resistant acne.▪ Culture and sensitivity testing should be reserved for treatment-refractory cases or cases indicative of secondary infection.

### 3.2. Supportive Care and Lifestyle Management in Acne Vulgaris

Recommendations 6–9 emphasize the role of supportive and adjunctive strategies in the management of acne, including patient education. Given that acne is a chronic inflammatory condition, setting realistic expectations through patient education is crucial to promote treatment adherence and long-term success [23]. Patients should be informed about the nature of acne, anticipated treatment timelines, and the important of continued maintenance therapy.

Lifestyle factors, such as diet, sleep patterns, stress levels, and cosmetic product use, are increasingly being recognized as modifiable contributors to acne. Comprehensive history taking, including an assessment of the body mass index, obesity, and related factors, should be taken into account when identifying patients suitable for lifestyle management.

Incorporating dermocosmetics (cosmeceuticals) into daily skincare routine, including cleansers, moisturizers, and sunscreens, can enhance treatment tolerability and efficacy. These adjuncts help maintain skin barrier integrity, reduce irritation from topical medications, and improve overall satisfaction [26].

Emerging adjuncts such as probiotics have demonstrated the potential to reduce acne lesions and inflammation by modulating the skin microbiome [48]. On similar lines, a 2023 systematic review found that antioxidant supplements such as zinc, vitamin C, and nicotinamide may support skin health in acne-prone individuals, although further high-quality studies are needed to confirm their clinical benefit [49].
▪***Key expert recommendations***▪Clinicians should consider dietary adjustments (particularly glycemic index), stress reduction, and adequate sleep on a case-by-case basis, emphasizing comprehensive history taking.▪The use of lipid-free, noncomedogenic skincare products is recommended to support therapeutic outcomes and minimize exacerbation.

### 3.3. Topical Therapy for Acne Vulgaris

*Topical retinoids:* Recommendations 10–20 focus on topical therapies for acne vulgaris. Topical retinoids are widely regarded as first-line treatment options for mild-to-moderate acne due to their comedolytic, anti-inflammatory, and preventive effects on lesion formation. Among them, adapalene 0.1% is most preferred for its favorable efficacy and tolerability profile [27]. In comparison, tretinoin, though effective, may cause photosensitivity, erythema, and irritation [50]. Consequently, adapalene is often the preferred choice for patients with sensitive skin. A head-to-head study comparing the efficacy and tolerability of adapalene gel 0.1% vs. tretinoin cream 0.025% in patients with mild-to-moderate acne vulgaris found that adapalene demonstrated superior tolerability, with fewer cutaneous side effects [51].

Tazarotene, a synthetic retinoid recommended for moderate to severe acne, is well tolerated, safe, and effective but is associated with dryness and peeling. Its once-daily use and low concentration (0.05% [50 mg/g]) make it suitable for use in large areas such as the trunk [52]. However, it is associated with local irritation, which may affect adherence [53,54].

Trifarotene (0.005% or 50 mcg/g) is a fourth-generation retinoid that has shown promise in treating acne and PIH, particularly when combined with an appropriate skincare regimen. Clinical trials have demonstrated significantly greater lesion count reductions compared to vehicles at 12 weeks. Over 90% of patients reported high satisfaction with trifarotene, noting less dryness and irritation [55].

Topical retinoids are effective as monotherapy for mild-to-moderate acne. A systematic review confirmed the overall superiority of retinoids over a vehicle in improving acne severity. Improvements in Investigator’s Global Assessment (IGA) and Investigator’s Static Global Assessment scores ranged from 24.1 to 28.8% and 13.3 to 17.3%, respectively (*p* < 0.001). While no significant efficacy differences were found between tretinoin and Tazarotene, tolerability varied widely. Tretinoin 0.05% was associated with adverse events (AEs) in 62% of patients compared with 19% and 40% with adapalene 0.1% and 0.3%, respectively. Notably, more patients reported tolerable AEs with adapalene than with tazarotene (55.4% vs. 24.4%, *p* < 0.0012) [27].

In summary, all topical retinoids are effective, but their selection should be tailored to the patient’s skin type, anticipated response, and tolerability profile. Adapalene offers the best balance between efficacy and tolerability, tazarotene provides slightly greater efficacy but with higher irritation potential, and tretinoin shows relatively rapid results with intermediate tolerability.

*Benzoyl peroxide:* Benzoyl peroxide (BPO) is effective as a monotherapy for mild acne at concentrations of 2.5% and 5%. It is suitable for use on both facial and truncal acne and is valued for its antimicrobial activity against *C. acnes*, as well as its comedolytic and anti-inflammatory effects. BPO use does not lead to anti-microbial resistance, making it a cornerstone in both initial and maintenance acne therapy [27].

*Topical antibiotics*: In a recent study, 2% ozenoxacin, which was approved in India in 2022, demonstrated superior efficacy over clindamycin in reducing acne lesion severity and associated symptoms (*p* < 0.05) [56]. Topical 4% minocycline has also demonstrated good efficacy in treating inflammatory lesions of moderate-to-severe non-nodular acne vulgaris [57,58]. Minocycline 4% gel, which was approved in India in 2022, showed significantly greater reductions in acne lesions (−87.8% vs. −63.59%; *p* < 0.001) and a higher IGA treatment success (73.9% vs. 46.7%; *p* = 0.015) compared to clindamycin 1% at 12 weeks, along with better tolerability parameters versus the clindamycin 1% arm, supporting its use in treating moderate-to-severe acne in the Indian population [59].

Topical dapsone (5% or 7%) offers good antimicrobial and anti-inflammatory properties and is effective as monotherapy or in combination with isotretinoin. It is generally well tolerated, though mild, transient adverse effects such as dryness or localized eczema may occur [29]. New hydrogel-based formulations have improved its usability despite the molecule’s poor water solubility.

Given increasing concerns about antimicrobial resistance, non-antibiotic alternatives are gaining traction and preference. Azelaic acid 20% is once such agent, demonstrating significant reductions in acne lesion counts and severity with a favorable safety profile. It is particularly suitable for individuals with skin of color or those intolerant to retinoids [60]. Metronidazole, typically used in rosacea management, may also be effective in cases of moderate acne vulgaris [61]. Both 0.75% and 1.0% formulations, when used daily, are well tolerated and beneficial for steroid-induced or rosacea-like acne presentations [62].

Topical salicylic acid gel is a comedolytic and anti-inflammatory agent widely used in the treatment of mild-to-moderate acne vulgaris. In a randomized study involving 500 patients with mild-to-moderate acne, 2% supramolecular salicylic acid gel showed a 51.01% regression or a marked improvement in acne lesions at 12 weeks compared to 43.1% improvement with adapalene gel (*p* = 0.0831), with a lower AE rate (0.40% vs. 0.80%) and significant cosmetic benefits, including improved pore appearance [63].

#### Combination Therapy

Although BPO monotherapy is effective, fixed-dose combinations, particularly with topical retinoids, offer superior efficacy and patient adherence. The combination of adapalene and BPO can show reductions in acne lesions by up to 70.2%, with mild, self-resolving adverse effects as per a systematic review [33]. Clinical studies have shown that this combination produces significant improvements in as early as one week and is more effective than either component used alone [64,65,66]. It is also preferred for long-term maintenance therapy [27]. For moderate-to-severe acne, adapalene 0.3% combined with BPO 2.5% has demonstrated sustained efficacy, with improvements in both active lesions and atrophic scars maintained over 48 weeks [67,68,69].

In patients with Grade 4 acne, a combination of nadifloxacin 1% and adapalene 0.1% has shown to reduce acne severity significantly within 5 weeks and is generally well tolerated [70]. The CACTUS trial demonstrated that the combination of 0.1% adapalene and 1% clindamycin was well tolerated and more effective than monotherapies for moderate acne [71]. Similarly, the combination of 1.2% clindamycin phosphate with 0.025% tretinoin gel proved superior to monotherapy [72]. A study comparing the efficacy and safety of topical combinations showed a greater reduction in total lesion count with the clindamycin 1% and BPO 2.5% combination, while nadifloxacin 1% with BPO 2.5% had a better safety profile, both with statistically significant results [73].

When combined with BPO 2.5%, nadifloxacin 1% showed comparable efficacy with clindamycin 1%, significantly reducing total, inflammatory, and noninflammatory lesions [74]. To limit antimicrobial resistance, both topical and systemic antibiotics should be restricted to short-term use, ideally not exceeding three months [44,58]. Since adherence to acne therapy is often hindered by skin irritation, selecting agents with a favorable safety profile is essential to optimize outcomes and reduce dropout rates [54].


***Key expert recommendations***
Adapalene 0.1% is the preferred choice of topical retinoid due to its favorable tolerability profile, followed by tretinoin 0.025% and adapalene 0.3%. Trifarotene, a fourth-generation retinoid, is effective and well tolerated for moderate facial and truncal acne, with additional benefits in treating PIH. For combination therapy, adapalene 0.1% remains the agent of choice, followed by adapalene 0.3% and tretinoin 0.025%. ▪Newer formulations of tretinoin, such as microsphere or microsponge delivery systems, may offer improved tolerability.▪The frequent application of a noncomedogenic moisturizer and short contact therapy may reduce irritation.▪Monotherapy with antibiotics is strongly discouraged to prevent the development of antimicrobial resistance.▪Topical antibiotics such as clindamycin, minocycline, nadifloxacin, and ozenoxacin may be used in combination therapy for acne for up to 12 weeks.

### 3.4. Systemic Therapy in Acne Vulgaris

Recommendations 21–28 address the role of systemic therapy in acne management, particularly for patients with moderate-to-severe disease, the involvement of large body areas (e.g., the trunk), or poor response to topical treatments. Systemic therapy is also indicated for patients with nodulocystic or scarring acne, adult-onset acne, or acne associated with significant psychological distress [75].

Oral isotretinoin is considered the treatment of choice for severe, recalcitrant, or scarring acne. It is typically initiated at a dose of 0.5 mg/kg/day and may be titrated up to 1 mg/kg/day if tolerated well [22].

Although highly effective, isotretinoin is associated with several dose-dependent side effects, including xerosis (dry skin), cheilitis, musculoskeletal discomfort (e.g., back pain), transient elevations in liver enzymes, dyslipidemia, thrombocytopenia, insulin resistance, inflammatory bowel disease, and rarely, psychiatric symptoms such as insomnia or depression. Hair loss has been reported in 3.2% of patients at 0.3 mg/kg/day, increasing to 5.7% at 0.5 mg/kg/day.

To minimize the risk of flare-ups during treatment initiation, a conservative starting dose of ≤0.5 mg/kg/day is recommended. A cumulative dose of 120 mg/kg is generally considered both efficacious and safe. In cases of significant adverse events, temporary dose adjustments or treatment interruption may be warranted [13].

*Oral antibiotics:* Oral antibiotics are indicated for patients with moderate-to-severe acne, particularly when inflammatory lesions are prominent or the acne is non-responsive to topical treatments. Their use should be restricted to a maximum duration of 12 weeks to minimize the risk of antimicrobial resistance. Importantly, systemic antibiotics must always be combined with topical agents (e.g., BPO or retinoids) to improve efficacy and further reduce resistance development [44].

*Tetracycline-class antibiotics*—including doxycycline and minocycline—are widely preferred due to their dual antimicrobial and anti-inflammatory effects. Doxycycline is preferred for moderate-to-severe acne, as it is effective and has the convenience of once-daily dosing (50–200 mg/day) for a duration of 6–8 weeks [76]. Minocycline is often favored for its better sebaceous gland penetration and fewer gastrointestinal disturbances than doxycycline [77]. Lymecycline is an optional antibiotic that can be considered, although its use is limited [78].

*Macrolide antibiotics:* Macrolides, such as erythromycin, azithromycin, and clarithromycin, are commonly prescribed for acne. However, increasing resistance to *Cutibacterium acnes* is a growing concern [79]. Azithromycin, often administered in a dosage of 500 mg thrice weekly, is reserved for patients intolerant to tetracyclines but carries a higher risk of resistance [44,77]. Treatment is usually limited to 12 weeks, with efforts to minimize duration whenever possible [80].

*Third-line and alternative therapies:* As a third-line therapy, trimethoprim–sulfamethoxazole is effective in acne treatment but carries the risk of rare, severe AEs, including Stevens–Johnson syndrome and toxic epidermal necrolysis [77]. It is generally reserved for cases where other antibiotics have failed.

*Combination therapy:* Combination therapies are often recommended to improve outcomes and reduce the development of resistance. Acne management guidelines suggest combining systemic therapies with topical treatments, such as BPO or retinoids [22].

Findings from a systematic review showed that combination therapy with a topical retinoid and an oral antibiotic resulted in significantly greater lesion count reduction compared with the vehicle treatment (64–78.9% vs. 41–56.8%; *p* < 0.001) [27].

As per a recent study, topical minocycline 4% in combination with oral isotretinoin was found to be more effective than oral isotretinoin in moderate-to-severe acne vulgaris [81].

The combination of benzoyl peroxide, a topical retinoid, and oral tetracycline demonstrated a treatment response rate ranging from 43% to 53%, indicating notable efficacy in the management of moderate-to-severe acne [82].

A prospective cross-sectional study found that a greater proportion of patients receiving combined oral and topical therapies achieved IGA scores of 0 or 1, indicating superior clinical improvement [83]. The concomitant use of topical benzoyl peroxide or a retinoid is recommended with systemic antibiotics for maintenance after completing antibiotic therapy [37].

*Hormonal therapy:* Acne in females often involves underlying metabolic or hormonal imbalances that may contribute to its persistence. A study of 135 Indian females with acne found that those with persistent acne exhibited significant clinical hyperandrogenism, PCOS, and hormonal imbalances, highlighting the need for endocrinological evaluation [46]. For hormonal acne in females, estrogen- and progestin-containing combined oral hormonal contraceptives (CoHCs) effectively treat acne due to their antiandrogenic properties and are suitable for patients seeking contraception [84].

Spironolactone is safe and effective for the long-term treatment of adult female acne, including truncal acne. The SAFA trial demonstrated that spironolactone (50 mg and 100 mg) was well tolerated with mild side effects. It is a viable alternative to oral antibiotics for females with persistent acne unresponsive to first-line topical therapies [85].

The addition of metformin can significantly reduce acne in patients with underlying metabolic or hormonal disorders, such as PCOS. Metformin may also be considered for patients who are non-responsive to standard acne treatments, particularly for those with insulin resistance or metabolic concerns [36].

Combining isotretinoin (0.5 mg/kg/day) with prednisolone (30 mg/day) should only be performed in the context of acne fulminans, and the experts gave a conditional recommendation for this [22].

*Special situations:* Intralesional triamcinolone injections (2.5 mg/mL–0.05 mL) may be considered for large acne lesions [22]. However, adverse effects, such as skin atrophy, were observed in fewer than 1% of patients [86].
▪***Key expert recommendations***▪Treatment with isotretinoin typically lasts 4–6 months, with dose adjustments based on clinical response and side effects.▪Topical therapies may be combined with systemic treatments; however, the usage of a similar class of topical and systemic agents is discouraged.▪To minimize the risk of resistance and side effects, oral antibiotics should be limited to 3 months.▪When treating adult acne in sexually active females, spironolactone can be combined with CoHCs.▪Experts provided a conditional recommendation supporting the use of oral corticosteroids, in combination with isotretinoin, for the medical management of acne fulminans.

### 3.5. Acne Management in Pregnancy and Lactation

Statement 29 gives recommendations on the medical therapies in pregnancy and lactation. Managing dermatologic conditions during pregnancy and lactation requires balancing treatment benefits with potential risks to the fetus or infant. Due to the lack of comprehensive guidelines on acne management in pregnancy and lactation, treatment must be individualized based on acne severity and the patient’s circumstances.

*Preconception considerations:* Although topical retinoids pose a low risk, they are best avoided during pregnancy as a precaution. Tretinoin is usually cleared from the body within a day in healthy, non-pregnant adults, though metabolism varies individually. Their use should be limited or discontinued before conception.

*Therapies contraindicated in pregnancy and lactation:*

Isotretinoin is strictly contraindicated due to its known teratogenic effects, and pregnancy should be avoided for six months after ceasing treatment. Individuals should also be counseled about the risks related to isotretinoin in pregnancy [87]. Spironolactone should also be discontinued at least 1 month to 6 weeks before conception due to its antiandrogenic effects. Additionally, dapsone and sulfamethoxazole are not indicated due to limited data regarding risks to the fetus [88]. Table 2 gives an overview of the drugs considered safe, contraindicated, or to be used with caution in pregnancy and lactation [88].

**Table 2:** Overview of drugs in pregnancy and lactation.

Drug	Route of Administration	Pregnancy	Lactation
Tretinoin	Topical	Contraindicated	Considered safe
Adapalene	Topical	Contraindicated	Use with caution
Benzoyl peroxide	Topical	Considered safe	Use with caution
Clindamycin	Topical and systemic	Considered safe	Considered safe
Salicylic acid	Topical	Considered safe	Considered safe
Dapsone	Topical	Not studied	Use with caution
Minocycline	Topical and systemic	Contraindicated	Considered safe for short-term use
Azelaic acid (<4%)	Topical	Considered safe	Considered safe
Isotretinoin	Systemic	Contraindicated	Contraindicated
Azithromycin	Topical and systemic	Considered safe	Considered safe
Erythromycin	Topical and systemic	Considered safe	Considered safe
Penicillin	Topical and systemic	Considered safe	Considered safe
Cephalosporins	Topical and systemic	Considered safe	Considered safe
Cotrimoxazole	Systemic	Contraindicated	Contraindicated
Spironolactone	Systemic	Contraindicated	Use with caution
Corticosteroids	Systemic	Contraindicated	Can be used; delay nursing by 3–4 h

▪***Key expert recommendations***▪Medical therapies contraindicated during pregnancy and lactation include topical and oral retinoids, tetracycline antibiotics, spironolactone, and oral contraceptives

### 3.6. Complications of Acne Treatment: PIH, PIE, and Acne Scarring

Recommendations 30–34 cover the management of PIH and acne scars, focusing on effective treatments for hyperpigmentation that often follows acne. PIH and PIE (also known as acne-induced macular erythema) are considered cosmetically unacceptable by patients and may add to the psychosocial burden in individuals with acne.

*Topical retinoids:* Topical retinoids are recommended as a first-line treatment for patients with acne-associated PIH. Tretinoin 0.025% cream and adapalene 0.1% gel show significant improvement in treating acne and PIH in patients with skin of color; however, tretinoin may cause irritation and inflammation. Adapalene 0.3% gel effectively treats atrophic acne scars and improves skin texture, with good tolerability [42].

*Skin-lightening agents*: Hydroquinone is a key skin-lightening agent that may be combined with retinoids. However, it should be applied specifically to the affected areas in acne-related PIH to prevent unintended hypopigmentation [89]. A formulation containing 3% tranexamic acid, 1% kojic acid, and 5% niacinamide was shown to be a safe and effective treatment for PIH, demonstrating significant improvement in hyperpigmentation at week 12 compared to baseline [90]. Topical azelaic acid 20% cream and 5% tranexamic acid, also valuable options, were compared in a single-blind randomized trial, demonstrated comparable but statistically significant reductions in post-acne hyperpigmentation index (PAHI) measurements at week 12 compared to baseline [91].

*Cosmeceuticals and adjunctive treatments:* Cosmeceuticals containing ingredients such as salicylic acid, alpha-hydroxy acids, and niacinamide offer benefits when used as an adjunctive treatment for acne management [16]. These ingredients enhance the overall tolerability and effectiveness of acne and PIH treatments.

*Sun protection and skincare:* Broad-spectrum sunscreens with ultraviolet (UV) and visible light protection are essential for preventing the worsening of hyperpigmentation, and proper counseling on their use enhances treatment outcomes. A comprehensive skincare routine, including moisturizers, cleansers, and UV protection with physical sunscreen, can be particularly helpful in managing acne-induced PIH [39].
▪***Key expert recommendations***▪Experts recommend topical retinoids as a first-line treatment for acne-associated hyperpigmentation, with adapalene 0.1% and 0.3%, tretinoin 0.025% and 0.05%, and trifarotene 50 mcg/g 0.05% being the preferred options for acne scar management.▪Adapalene 0.3% + BPO 2.5% gel is particularly effective for treating atrophic acne scars and improving skin texture, with good tolerability.▪Experts advised against the use of Kligman’s formula and topical steroids for PIH.

### 3.7. Retinoid-Induced Dermatitis

Statements 35–36 elaborate on retinoid-induced dermatitis. Retinoids are highly effective in treating acne and other dermatological conditions, but they may cause dose-dependent irritation in some patients, a condition known as retinoid dermatitis. Symptoms include erythema, pruritus, burning, xerosis, and desquamation [92].

*The importance of skin tolerability:* Ensuring optimal skin tolerability is essential for successful acne treatment. The CHARISMA study—a retrospective, multicenter study in India, demonstrated that adding a moisturizer with broad-spectrum sunscreen to acne treatment enhanced skin tolerability and patient satisfaction [93].
▪***Key expert recommendations***▪Mild irritation or dryness with retinoids, especially during the initial weeks or in dry, cold weather, may occur.▪Experts recommend the regular use of a noncomedogenic moisturizer and a gentle cleanser to manage irritation and prevent dryness, maintain skin hydration, and protect the skin barrier.▪A gentle, nonirritating cleanser should be used to prevent further irritation to the skin.▪Patients should be educated on the proper application of retinoids, including using a minimal amount of product to avoid excessive irritation and enhance treatment adherence.▪To minimize the risk of irritation, experts recommend starting with a low concentration of retinoids and adopting an alternate-day dosing schedule for the first 2 weeks.

### 3.8. Maintenance Therapy

Individuals with frequent acne relapses after treatment are candidates for maintenance therapy. A fixed combination of topical adapalene and benzoyl peroxide is recommended. If it is not tolerated or contraindicated, adapalene, azelaic acid, or benzoyl peroxide alone can be used. The need for maintenance therapy may be after 12 weeks [94].

### 3.9. Algorithm for Diagnosis and Medical Management of Acne

**Figure 1:**
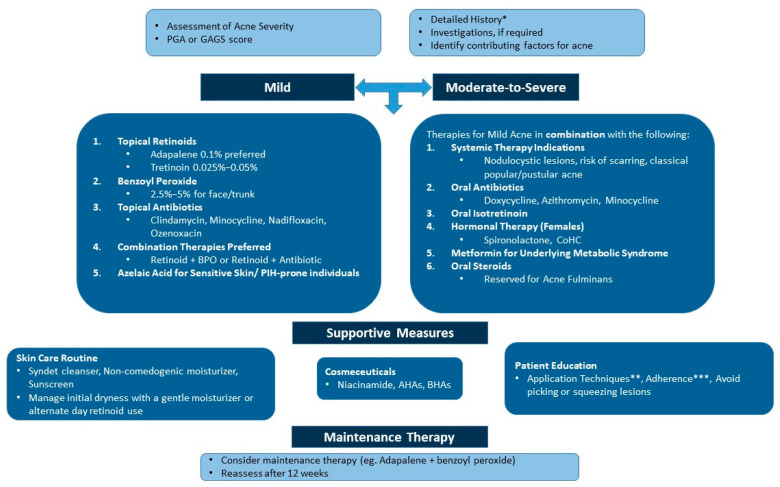
An algorithm for the diagnosis and medical management of acne. AHA: alpha-hydroxy acid; BHA: beta-hydroxy acid; BPO: benzoyl peroxide; CoHC: combined oral hormonal contraceptive; GAGS: Global Acne Grading System; PGA: Physician’s Global Assessment; PIH: postinflammatory hyperpigmentation. * These can range from lifestyle factors, such as nutrition, stress, sleep, etc., to hormonal factors. ** A pea-sized amount of retinoids at night and the quantity of sunscreen to be applied. *** Encourage the consistent use of treatment modalities. Maintenance therapy is crucial after clearance.

**Figure 2:**
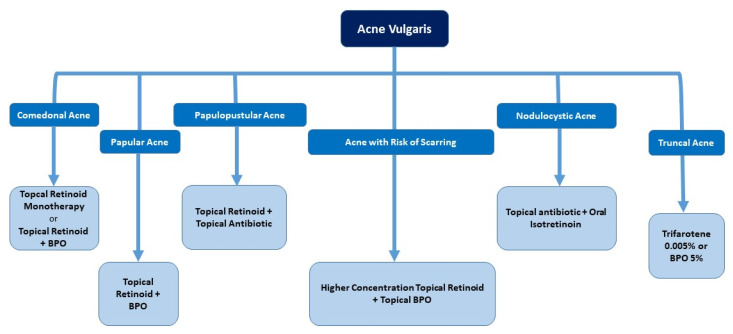
Retinoid-based therapies as per acne lesions. BPO: benzoyl peroxide.

Building on the above insights, the expert panel developed a practical algorithm for managing acne in the Indian population, emphasizing the need for tailored treatment approaches to balance effectiveness and skin comfort. Figure 1 provides a comprehensive diagnosis and management algorithm for acne based on expert consensus. Figure 2 gives a straightforward approach to choosing retinoid-based therapies based on acne lesions.

## 4. Methodology

The consensus recommendations were developed through a structured, evidence-based process led by a panel of 14 dermatology experts, each with over 15 years of clinical experience and a strong record of academic contributions. A comprehensive literature search was conducted across PubMed, Scopus, and the Web of Science to identify relevant studies on acne management, including general treatment strategies and special considerations during pregnancy and lactation. Studies published within the previous 5 and 10 years were considered eligible for inclusion.

The search strategy included both Medical Subject Headings and free-text keywords included “acne”, “acne treatment”, “medical management”, “retinoids”, “topical”, “oral antibiotics”, “systemic therapy”, “pregnancy”, “lactation”, “PIH”, “retinoid-induced dermatitis”, “cosmeceuticals”, and “dermocosmetics.” Relevant articles were selected following full-text screening to ensure a robust evidence base for discussion.

The next step involved the formulation of 61 key clinical statements, categorized into seven thematic domains:

(I) Investigations for acne vulgaris;
(II) Supportive care and lifestyle management in acne vulgaris;
(III) Topical therapy in acne;
(IV) Systemic therapy in acne;
(V) Considerations in pregnancy and lactation;
(VI) PIH and acne scarring;
(VII) Retinoid-induced dermatitis.

Each statement was graded using the Oxford Centre for Evidence-Based Medicine Levels of Evidence presented in Table 3.

The consensus process utilized a modified Delphi technique, with two rounds of anonymous voting conducted on a virtual platform. The responses were collected anonymously. Experts consented to the aggregate analysis of their responses. Consensus was defined as ≥75% agreement on a 5-point Likert scale. Statements were then categorized into positive recommendations, where ≥75% of participants agreed or strongly agreed, or negative recommendations, where ≥75% disagreed or strongly disagreed. Statements with 50–74% agreement were considered to have near consensus, while those with <50% agreement were classified as having no consensus. Statements not achieving 75% concordance were revisited for further discussion.

## 5. Conclusions

This consensus provides comprehensive, evidence-based recommendations for diagnosing and managing acne vulgaris in India. The expert panel has established practical guidance for dermatologists and general practitioners by addressing key clinical domains, including baseline investigations, treatment strategies, skincare, and special considerations, such as pregnancy and PIH. Additionally, the proposed algorithm offers a structured approach to optimizing acne management, ensuring better patient outcomes through individualized and informed decision-making.

## Figures and Tables

**Table 1:** Expert recommendations on the diagnosis and management of acne in India.

Statements		Evidence	Grading of Evidence	Level of Agreement %	Recommendation
**I. Investigations in Acne Vulgaris**					
1.	Blood tests should be done routinely before starting isotretinoin for acne.	[19]	2b	77%	Positive
2.	Minimum blood tests should include serum triglycerides, ALT, and pregnancy tests for females of reproductive age.	[20]	3b	85%	Positive
3.	Psychological assessments should also be incorporated before the commencement of isotretinoin.	[21]	1a	85%	Positive
4.	Blood tests are routinely required to start oral antibiotics for acne vulgaris.	[22]	1a	85%	Negative
5.	Hormonal blood investigations and pelvic ultrasounds should be routinely conducted in female patients with acne vulgaris, especially in cases of irregular menstrual cycles, suspected or known PCOS, or signs of hyperandrogenism.	[23]	3b	92%	Positive
**II. Supportive Care and Lifestyle Management in Acne Vulgaris**					
6.	Patient education should emphasize the chronic nature of acne, treatment expectations, and the importance of adherence.	[24]	1a	100%	Positive
7.	Cosmeceuticals, including cleansers, moisturizers, and sunscreens, can be incorporated into the medical management of acne vulgaris to streamline a daily routine.	[25]	1b	85%	Positive
8.	Lifestyle and dietary modifications should be included as part of the general approach to managing acne vulgaris.	[26]	2a	100%	Positive
9.	Patients should use syndet/lipid-free cleansers and avoid moisturizers and sunscreens that are oil-based and comedogenic.	[26]	2a	100%	Positive
**III. Topical Therapy**					
10.	Topical retinoids should be the first-line treatment for all patients with mild-to-moderate acne vulgaris, in the absence of contraindications.	[27]	1a	100%	Positive
11.	The most preferred topical retinoid for acne vulgaris is adapalene 0.1%, followed by tretinoin 0.025% and adapalene 0.3%.	[27]	1a	85%	Positive
12.	BPO can be used as a monotherapy for mild facial acne and truncal acne with a preferred frequency of once a day, preferably in the evening.	[27]	1b	85%	Positive
13.	BPO 2.5%, and to a lesser extent BPO 5%, can be used for acne.	[27]	1b	92%	Positive
14.	Topical antibiotics like clindamycin, minocycline, nadifloxacin, and ozenoxacin can be used for the medical management of acne for a period of 3 months or less as a combination therapy. Avoid monotherapy with topical antibiotics.	[28]	1b	92%	Positive
15.	Topical dapsone can be recommended for inflammatory acne.	[29]	1a	92%	Positive
16.	Topical metronidazole can be used to treat acne associated with rosacea and steroid-induced acne.	[30]	1b	77%	Positive
17.	Topical azelaic acid is suitable for patients intolerant to other topical therapies and those concerned about PIH.	[31]	1b	77%	Positive
18.	Retinoids should be combined with BPO or antibiotics in cases with papulopustular lesions (Grade II–III).	[32]	1b	92%	Positive
19.	A combination of BPO with adapalene 0.1% or 0.3% is recommended over antibiotics in acne treatment.	[33]	1a	77%	Positive
20.	The preference of topical retinoids for combination is adapalene 0.1%, followed by adapalene 0.3% and tretinoin 0.025%.	[27]	1a	92%	Positive
**IV. Systemic Therapy**					
21.	Systemic therapy can be started in patients with	[28]	1b	85%	Positive
	a. Moderate-to-severe acne vulgaris				
	b. Acne resistant to topical treatments				
	c. Acne with a risk of scarring				
	d. Acne involving large body areas				
	e. Hormonal acne in females				
	f. Acne causing severe distress or psychological impact				
	g. Adult acne				
22.	Oral isotretinoin can be started in the absence of contraindications; the initial dose is 0.25–0.5 mg/kg/day, which can be uptitrated as per tolerability to 1 mg/kg/day.	[22]	1a	92%	Positive
23.	Risk of scarring may be increased by	[34]	1a	100%	Positive
	a. Nodulocystic or severe acne				
	b. Delay in seeking treatment				
	c. Picking or squeezing of the acne lesion				
	d. Family and past history of acne scarring				
24.	Recommended oral antibiotics for the management of acne vulgaris are doxycycline, azithromycin, and minocycline. Optional antibiotics include lymecycline and cotrimoxazole.	[28]	1b	77%	Positive
	Oral antibiotics can be given once daily for up to 3 months or less in combination.				
25.	Preferred hormonal therapies for adult females with acne vulgaris include spironolactone and CoHC pills.	[35]	1a	92%	Positive
26.	Metformin can be added as an adjunct for patients with underlying metabolic syndrome.	[36]	1a	77%	Positive
27.	Oral corticosteroids (combined with isotretinoin) can be used for the medical management of acne fulminans.	[22]	1a	46% agree, 46% neutral	Conditional
28.	Intralesional triamcinolone can be used to treat nodulocystic acne lesions.	[22]	1a	92%	Positive
**V. Considerations in Pregnancy and Lactation**					
29.	The medical therapies contraindicated in pregnancy and lactation include topical and oral retinoids, topical and oral tetracycline antibiotics, spironolactone, and oral contraceptives.	[37]	1a	92%	Positive
**VI. PIH and Acne Scarring**					
30.	Topical niacinamide, AHAs, and BHAs may be recommended for acne.	[38]	1a	85%	Positive
31.	PIH can be prevented by limiting the inflammation with timely and appropriate medical management in acne vulgaris and by using a noncomedogenic broad-spectrum sunscreen.	[39]	1a	85%	Positive
32.	Tretinoin, kojic acid, niacinamide, hydroquinone, and azelaic acid are preferred in the management of PIH.	[40]	1a	77%	Positive
33.	Kligman’s formula and topical steroids should be avoided.	[40]	2b	100%	Positive
34.	Among retinoids, adapalene 0.1% and 0.3%, tretinoin 0.025% and 0.05%, and trifarotene 0.05% are useful in the medical management of acne scars.	[41]	1b	77%	Positive
**VII. Retinoid-Induced Dermatitis**					
35.	A temporary reduction in the dose, duration, and frequency or discontinuation of topical retinoids can mitigate retinoid-induced dermatitis.	[42]	1b	100%	Positive
36.	A gentle cleanser and noncomedogenic moisturizer help in the prevention and management of retinoid-induced dermatitis.	[25]	1b	100%	Positive

AHA: alpha-hydroxy acid; ALT: alanine aminotransferase; BHA: beta-hydroxy acid; BPO: benzoyl peroxide; CoHC: combined oral hormonal contraceptive; PCOS: polycystic ovary syndrome; PIH: postinflammatory hyperpigmentation.

**Table 3:** Oxford level of evidence [95].

Level of Evidence	Therapy/Prevention/Etiology/Harm	Prognosis
1a	Systematic review (with homogeneity) of RCTs	Systematic review (with homogeneity) of inception cohort studies; clinical decision rule validated in different populations
1b	Individual RCT (with narrow CI)	Individual inception cohort study with >80% follow-up; clinical decision rule validated in a single population
1c	All or none	All or none case series
2a	Systematic review (with homogeneity) of cohort studies	Systematic review (with homogeneity) of either retrospective cohort studies or untreated control groups in RCTs
2b	Individual cohort study (including low-quality RCTs, <80% follow-up)	Retrospective cohort study or follow-up of untreated control patients in an RCT; derivation of clinical decision rule or validated on a split-sample only
2c	“Outcomes” research and ecological studies	“Outcomes” research
3a	Systematic review (with homogeneity) of case–control studies	–
3b	Individual case–control study	–
4	Case series (and poor-quality cohort and case–control studies)	Case series (and poor-quality prognostic cohort studies)
5	Expert opinion without an explicit critical appraisal or based on physiology, bench research, or “first principles”	Expert opinion without an explicit critical appraisal or based on physiology, bench research, or “first principles”

CI: confidence interval; RCT: randomized controlled trial.

## Data Availability

The data supporting this study’s findings are available from the corresponding author upon reasonable request.
